# Teaching Children’s Motor Skills for Team Games Through Guided Discovery: How Constraints Enhance Learning

**DOI:** 10.3389/fpsyg.2021.724848

**Published:** 2021-12-10

**Authors:** Karl M. Newell, Inez Rovegno

**Affiliations:** ^1^Department of Kinesiology, University of Georgia, Athens, GA, United States; ^2^College of Education, University of Alabama, Tuscaloosa, AL, United States

**Keywords:** game skills, tactics, guided discovery, constraints, dynamical systems, affordances, expert teachers

## Abstract

In this paper we examine the role of instructional strategies as constraints within a discovery learning framework for the teaching of open skill team ball games to elementary school-aged children. The cohesive and adaptive integration of constraints (individual, environment, and task) by practitioners of movement and physical activity (instructor, teacher, coach) is proposed as the pathway to exploiting the effectiveness of guided discovery learning. The qualitative analysis of the practical instantiations of this framework by expert teachers is examined with respect to the learning of open skill team invasion games (e.g., basketball, soccer). The primary constraints to action in this learning-teaching developmental framework are coordinated so as to keep the self-organization of skill development (movement pattern and tactics) continually evolving, while preserving the child’s motivation and enjoyment for the expanding repertoire and performance capacity of his/her perceptual-motor skills. In this open skill and elementary school age-related context, generality *and* specificity are both necessary and complementary in the expression of task, skill and practice influences on motor learning and performance.

## Introduction

The progression of physical growth and movement patterns from early through late childhood reveals continuity and discontinuity in the development of functional competences in perceptual-motor skills (Bruner, 1961; Connolly, 1970; Muchisky et al., 1996; Savelsbergh et al., 1999; Adolph and Hoch, 2019). This evolving movement action repertoire is reflected in children’s capacity to learn by adulthood what seems to be an unlimited number of different perceptual-motor skills that can be performed in a variety of contexts. Moreover, there are occasionally individual children within a cohort age group that have prodigy-like elite levels of skill in a single activity, game or sport.

A long standing position in motor development is that children’s core developmental activities emerge (approximately 2 – 18 years) from the fundamental infant motor development sequence (approximately birth – 2 years) (Newell, 2020). The core developmental skills have been viewed as consequences of generality from the transfer of infant fundamental skills that have been assumed to in turn be antecedents to the development of the broad range of perceptual-motor skills in context. These include the activity contexts of play, games and sport (Wickstrom, 1977; Seefeldt, 1980; Wormhoudt et al., 2018), the broader action contexts of self-help skills and activities of daily living (Sugden and Wade, 2013), together with movement activities in expressions of art, music, and work.

The focus of this paper is the generalization of core developmental skills to become the open skills of team ball games progressing to the use of those skills and tactics in modified games. The developing movement patterns of elementary school aged children were analyzed at the ages that junior sport participation begins. This population was studied to acquire descriptive accounts that guide teaching of developmental changes in movement patterns of open game skills and rudimentary tactics together with the impact of individual, task, and environmental constraints on children’s learning of skills and tactics in context. School settings serve children with a wide range of developmental levels from beginners through highly competent players and the children typically have greater cultural and economic diversity than junior sport settings. Thus, school settings can provide a broad population cohort skill base for acquiring information on nurturing the development of game skills and game play.

A framework for learning-teaching is necessarily going to be a wide ranging if not an all-encompassing enterprise that is more comprehensive than we can approach here. In the remainder of this introduction we outline the key constructs at work in our approach to children’s discovery learning of perceptual-motor skills.

### School System Physical Education

School system physical education has long been promoted as the vehicle for the development of a broad base of skilled movements, dispositions, and knowledge that would enable students to enjoy a lifetime of meaningful, healthful physical activities (Williams, 1930; Hulteen et al., 2018). Nevertheless, the drawing on theories of learning to guide analysis of the learning and teaching of perceptual-motor skills in physical education and junior sport has not been as influential a perspective as might be anticipated (Rink, 1999; Quennerstedt and Maivorsdotter, 2017). Rather, the core developmental perceptual motor skills have primarily been associated with a narrow set of didactic methodologies focusing on children learning a rigid set of techniques as performed in closed environments by skilled athletes (Kirk, 2010; Rudd et al., 2021).

In this apparent standard orientation to teaching, the student is portrayed as *acquiring* behaviors or information received from the teacher or coach as provider (Quennerstedt and Maivorsdotter, 2017). Challenging this narrow approach are theoretically based curricular frameworks to learning *and* teaching, including teaching games for understanding, the movement or skill theme approach and, non-linear pedagogy (Logsdon et al., 1977; Almond, 1986; Kirk and MacPhail, 2002; Chow et al., 2016; Rovegno and Bandhauer, 2017; Renshaw et al., 2019; Graham et al., 2020).

Our context for observation and analysis is research on expert teachers of the movement approaches at the elementary school level. The movement approach began in England with the work of Laban (1948) and was developed primarily by female physical education college teachers into Educational Dance (Russell, 1958; Preston-Dunlop, 1963), Educational Gymnastics (Morison, 1960; Mauldon and Layson, 1965), and Educational Games (Mauldon and Redfern, 1969). The child centered movement approach was brought to the United States and Canada in the 1960s and further developed (see for example, Stanley, 1969; Logsdon et al., 1977; Mitchell et al., 2003; Rovegno and Bandhauer, 2017; Graham et al., 2020).

From the 1960s both in Britain and the United States the movement approaches were and remain learner-centered. Teachers designed tasks based on students’ capabilities and included student decision making thus enabling the accommodation of individual differences. Students, for example, made decisions about what movements and movement patterns to explore and practice, what movements to include in dance and gymnastics sequences, and their game design including game rules, procedures, equipment, and goals. Tasks typically constrained students to explore a broad concept such as balancing in gymnastics or a skill and concept from the Laban framework (see Table 1) such as in games dribbling on different pathways. Student autonomy was valued. This is in contrast to learning only those skills prescribed by the teacher performed in standard ways using standard techniques of highly skilled individuals.

**TABLE 1:** The Laban (1948) Framework Adapted for Educational Games.

Body: core developmental skills for games	Space movement elements
Locomotor skills: running, sliding,	• Areas: personal, general
jumping,	• Levels: high, medium, low
Manipulative skills:	• Pathways on the ground: straight, curved, zigzag
• Throwing overhand and underhand	• Pathways of balls in the air: straight, curved
• Passing	• Directions: forward, backward, sideways, upward, downward, diagonal
• Striking	• Extensions: near, far
• Volleying	
• Kicking	
• Dribbling	
• Catching	
• Receiving ball with stick or feet	
• Carrying	
Non-locomotor skills: pivoting, alert ready position, stretching, curling, twisting,	

**Effort movement elements**	**Relationship movement elements**

Speed	Body/body parts to equipment (e.g., rackets, balls, bats):
• Fast—slow	• In front of, behind, to the side of
• Accelerate—decelerate	• Over, under
Force:	Individuals and groups within game situations:
• Strong—light	Defensive tactics (examples):
• Producing—receiving	• Denying space
Use of space: direct—indirect	• Covering space
Flow:	• Gaining possession and intercepting
• Bound—free	• Marking: ball side/goal side
• Continuity of flow	• Backing others up
In relation to performance techniques:	• Shifting quickly to attack
• Using appropriate amounts of force and muscle tension	Offense tactics (examples):
• Using appropriate amounts of space	• Dribbling to avoid defenders
• Using appropriate amounts of speed	• Cutting into appropriate open space
• Controlling the flow of movement	• Sending lead passes
	• Creating space for self or others
	• Supporting the person with the ball
	• Hitting to open space
	• Making the defense shift
	• Shifting quickly to defense
	Game structures: rules, boundaries, consequences, scoring goals, scoring systems

*Source: Adapted from Barrett (1984a) and Rovegno and Bandhauer (2017).*

### Perceptual-Motor Learning and Development

Our theoretical approach to learning begins with motor development as it did with the movement approach in the United States from the 1960s on. A motor developmental perspective views development as related to the practice of core skills rather than being age-determined. Individual capabilities are recognized and accepted. Motor development research on the core developmental skills described qualitative non-linear changes in the movement patterns of body segments and/or phases of the skill (e.g., take off, flight, and landing of a standing long jump) (Wickstrom, 1977; Roberton and Halverson, 1977). Resulting from the relations among individual, task, and environmental constraints (Newell, 1986) these qualitative developmental changes emerge. The sequence of changes from immature to the mature patterns of skills guides teachers’ observations and interpretations of children’s responses and decisions as to whether the task and environment were working or needed modification to meet the goals of the lesson (Barrett, 1977).

In addition to developmental theory, our approach is grounded in the ecological approach to perception and action (Kugler et al., 1980, 1982; Turvey, 1990; Warren, 2006), the related domain of coordination dynamics (Kelso, 1995), with an emphasis on the role of constraints in movement coordination and control (Newell, 1986). In the constraints’ framework learning is an emergent feature of the search for the solution to harness the multiple degrees of freedom in the realization of a task goal (Bernstein, 1967; Newell et al., 1989). The ecological approach draws on J. J. Gibson’s (1979) theory of direct perception and the concept of affordances for the role of information in action (Turvey, 1992, Warren, 2006). In Gibson’s view, affordances are possibilities or opportunities for action and the discovery of their meaning by the individual is a hallmark of perceptual-motor development (E. J. Gibson, 1982; Adolph and Hoch, 2019). The ecological approach to perception maps to the related movement emphasis of Bernstein’s (1967) biophysical account of movement coordination of the multiple degrees of freedom (DF) as the foundation of motor development.

It is with this background to the embedded constructs of coordination, control and skill (Newell, 1985; Newell and Pacheco, 2019; Newell and Liu, 2020) that the role of task constraints in the context of the functional union of the individual and environmental affordances is viewed as fundamental in the self-organization of perceptual-motor development (Newell, 1986, 1991, 1996; Button et al., 2021). Constraints are foundational in the development of the generality *and* specificity of the open perceptual-motor skills of team games (Fischer and Farrar, 1987). Indeed, the nature of open skill games, aside from their relative degree of unpredictability (Poulton, 1957), is that they have a generality requirement (usually implicit) of a “set” of individual skills that are needed to be performed sufficiently well, sufficing (Simon, 1956) *and* a specificity requirement for sufficient expertise in particular individual skills of the game in question. This perspective on the open skills of games is pursued through three intertwined categories of observation and analysis of the development of young children’s perceptual motor skills, namely: task, skill level, and the practices of movement in context.

### Discovery Learning

The role of constraints is central within guided discovery teaching by expert teachers well-versed in guided discovery strategies. Their knowledge and careful design of tasks and the environmental constraints offer practitioners and coaches valuable information about qualitative changes in the movement patterns of skills resulting from constraints and guided discovery. These approaches to motor learning imply a shift from a teacher-centered approach in which the teacher makes all or most of the decisions about content, tasks, and goals to a learner-centered approach in which students make decisions about content, goals, and movement patterns and identify problems and search for solutions the environment affords. The search for solutions requires learners to explore multiple possibilities and engage in discovery learning guided by the teacher. They might search for specific techniques for movement patterns that work for the task set or discover different ways to vary or generalize the coordination pattern in relation to broader physical activity contexts in games (Vereijken and Whiting, 1990; Chow et al., 2016). Rather than the practitioner informing students what movements to perform, what techniques to use, and the specific outcome sought, the children search the perceptual-motor workspace in relation to their individual capabilities and what the environment affords to discover the emergence of movement patterns or activities that they practice (Newell et al., 1989; Newell, 1996; Adolph et al., 2011).

Discovery learning, also referred to as inquiry-oriented learning, is used in a range of subject matters supported by multiple theoretical frameworks. Consistent across school subject areas is the large body of research showing discovery learning needs to be carefully guided by the teacher to work well (Bruner, 1961; Vereijken and Whiting, 1990; Darling-Hammond et al., 2020). Teacher guidance includes scaffolding, teachers eliciting explicit information from students or providing explanations themselves, feedback for revising work, and guidance through questioning (Mayer, 2004; Alfieri et al., 2011; Osher et al., 2016; Cantor et al., 2018; Darling-Hammond et al., 2020). In our perspective, the core assumptions of discovery learning are built on: (a) the essential nature of self-organized search behavior by the learner; b) exploration of the environment by the learner; and c) the explorations and decisions in activity are child-centered rather than the development of skilled adult performance techniques prescribed by the teacher.

Discovery learning approaches to children’s motor learning can be difficult to implement and practitioners can develop misconceptions about them (Rovegno, 1992, 1993; McCaughtry and Rovegno, 2003; Chen, 2004). Most prominent among the misconceptions of discovery learning is that the instructor does not inform the children in any way as to what to do. Related misconceptions are that discovery tasks do not relate to skill development in sport, tactics are not taught, every movement the child produces is acceptable and positive, and there is no need to tell the children anything because they will perceive the task-relevant information through exploration. However, like other forms of teaching, guided discovery learning *requires* teacher intervention but in a way that channel’s the child’s search for a task relevant movement solution (Darling-Hammond et al., 2020).

There has been limited experimental study of the discovery learning approach in Physical Education in spite of a number of endorsements from within that community. An early experimental study of discovery learning compared a discovery strategy with a guided discovery strategy, and a strategy that combines these approaches (Singer and Pease, 1976). It was found in a computer managed task for manipulating objects, that the guided discovery and combination groups performed significantly better on one task with the discovery group scoring better on the retention test. Mosston’s (1966) divergent discovery style was also compared with a command/practice combination style and the divergent discovery group scored higher on producing divergent movement patterns (Cleland, 1994).

### Invasion Games

In the later sections of this paper we examine outcomes from studies of children (aged 7–10 years) learning the open skills of invasion games, mostly variations of basketball, and when ready progressing to game-like tasks and modified games with teachers applying constraints in an organized and progressive way to guide discovery (Rovegno and Bandhauer, 2017). The small group team game context has not been studied as extensively as the learning of individual closed movement skills in both children and adults, although there is the beginning of an experimental agenda with primarily adult performers on the acquisition of game tactical strategies (Araújo et al., 2004, 2009; Chow et al., 2016; Renshaw et al., 2019).

The contextual focus here is the learning-teaching research program of Rovegno and colleagues who observed elementary school children learning open skills from invasion ball game situations from expert teachers using a movement approach (Chen and Rovegno, 2000; Rovegno et al., 2001; Chen et al., 2003, 2012). Invasion games are one of four game categories based on tactical similarities (Mauldon and Redfern, 1969; Ellis, 1983; Almond, 1986; Oslin and Mitchell, 2006). Invasion games include basketball, team handball, football, field hockey, rugby, water polo, etc. in which both teams play in (invade) the same space. The other three categories are net/wall games, target games, and field games (e.g., cricket, softball, etc.).

The learning-teaching framework was examined through qualitative analysis of the integrated and progressive manipulations of individual, task, and environmental constraints and the impact on qualitative changes in children’s movement patterns. The analysis of constraints facilitated understanding the role of the task and environment set by the instructor to develop inquiry-oriented lessons through questioning, manipulating and modifying guided discovery tasks as needed, together with augmented information and feedback typically given in the form of questions or problems to solve (Newell, 1991, 1996; Newell and Ranganathan, 2010). Open skills and invasion games have received little to no research emphasis in motor learning and development with the majority of studies examining the learning and performance of individual closed skills, where the environment is highly predictable (Poulton, 1957).

## Movement Coordination, Control and Skill

The coordinative structure theory (Kugler et al., 1980) provided a background framework for the embedded constructs of coordination, control and skill and, in doing so, provided a potentially unifying framework to address core issues in what were then the largely separate domains of motor development, motor learning and motor control (Newell, 1985, 1986). Coordination is the function that constrains the potentially free variables into a behavioral unit. Control is the process by which parameter values are mapped to the function. Skill reflects the degree to which optimal values are related to the controlled variables. Coordination, control and skill are then in this framework embedded rather than independent processes.

### Constraints on Movement Coordination and Control

The coordinative structure theory (Kugler et al., 1980) gave emphasis to constraints as opposed to the traditions of prescriptions (e.g., motor programs) in motor learning and control (Turvey et al., 1978; Kelso, 1981). The theory characterizes the role of dynamics in the relations among the individual with the environment in pursuit of a task goal. From this general background, task constraints were integrated with those of the individual and environment to form a collective of constraints that coalesce to induce task-relevant movement forms for action (Newell, 1986).

**FIGURE 1:**
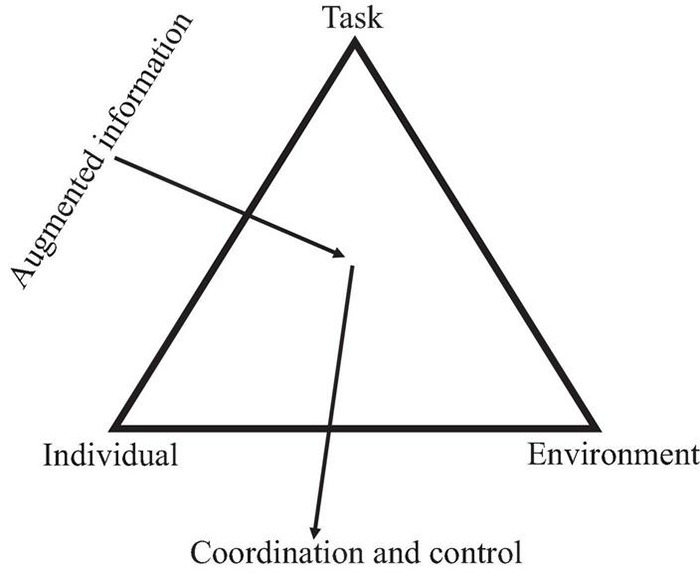
Constraints on the development of coordination (adapted from Newell, 1986).

In brief, constraints may be viewed as boundaries or features that limit motions of the entity under consideration. Constraints exist at various levels of analysis of the individual’s (or individuals’ as in team ball games) interaction with the environment and can be time dependent or time independent. Figure 1 shows a schematic of the general framework of constraints for movement and action and how augmented information in the form of instructions (e.g., feedback and feedforward) from an instructor can be considered coherently within this view (adapted from Newell, 1986).

Some basic experimental examples of the theoretical role of constraints and their interaction in motor development and learning were provided in Newell (1986). This framework has since been enriched by its integration into motor development (Haywood and Getchell, 2020) and motor learning (Button et al., 2021) together with its relevance in the more specialized sport, rehabilitation and pedagogy domains. Adolph and Hoch (2019) have shown how infant motor development is embedded in individual, task, and environmental constraints that create opportunities for possible actions with development expanding these opportunities. The cohesive and adaptive manipulation of constraints (individual, environment, and task) by practitioners of movement and physical activity (teacher, coach, therapist) is viewed here to be the instructional pathway to facilitating and exploiting the effectiveness of guided discovery learning.

### Adaptive Task Dynamics and Guided Discovery Learning

In motor learning and development it is recognized that a significant amount of practice over a substantial duration of time is typically required to realize a high level of performance outcome in a perceptual-motor skill (Ericsson et al., 1993). Indeed, in this view deliberate practice is the single most important variable supporting the acquisition of skill although its mode of implementation is typically different than that of discovery learning in the early stage of learning a skill. And, this leads to the related and common assumption that it takes longer practice time to learn more difficult motor tasks. This is a generalization that may not uniformly hold, however, in that individuals can have a special unique set of abilities (intrinsic dynamics in the coordination dynamics framework – Kelso, 1995) that map in a compatible way to particular tasks being learned rather than others. Moreover, certain kinds of motor tasks are more difficult to learn than others because a transition or qualitative change is required in the dynamics that the learner is not immediately able to perform (Newell, 1985; Zanone and Kelso, 1992; Newell and Liu, 2020). These features invite the proposal that ‘change’ in motor learning and development needs to be considered relative to the individual learner or group of learners rather than solely in the external frame of reference of the task goal.

Newell et al. (2009) outlined an approach to determining the *duality* of the difficulty of the motor task and the skill level of the individual (see also Liu et al., 2010). It was recognized that fixed external task criteria may not drive sufficiently the full potential of change in the dynamics of a system so as to reveal its universal properties. To overcome this limitation, a variant of “adaptive learning” (Proctor and Dutta, 1995) was proposed in which the experimenter/teacher continuously adapts the environmental conditions of the task so as to “keep” the learner at the critical point of the transition or in the developmental view at the edge of their current “zone of development.” The overall goal is to match the environmental demands to the skill level of the learner to preserve discovery learning and qualitative changes in movement patterns over changing time scales.

The roller ball task is especially suited to this adaptation protocol because it has the continuously varying control parameter of initial ball speed that is strongly correlated (inversely) with task difficulty across participants’ skill levels (Liu et al., 2006, 2010, 2019). Thus, task difficulty can be calibrated to an individualized skill level where the conditions can be tuned to produce a range of outcomes from 0 to 100% success. In Newell et al. (2009) it was proposed from elaborations of self-organized criticality theory that setting the initial conditions so that the learner on an individual basis has a 50% probability of success would maximize the learning rate (Bak, 1996). In this view, success in transfer depends on the performer having considerable practice operating at or near a critical point of a more difficult criterion.

This adaptive protocol can be modified so that it is the learner rather than the instructor that actively induces self-organization to the initial conditions (e.g., ball speed in the roller ball task) that can be changed in an adaptive way in terms of their perception of system stability/instability and performance level. This form of self-organization is a reflection of self-discovery and readily available to be observed in both children’s games or an individual child’s deliberate practice for the acquisition of a specific skill. Liu et al. (2012) provided evidence in adults for the unsupervised benefits of self-discovery learning through the adaptive setting of initial ball speed conditions in the roller ball task.

## Teaching for Guided Discovery Learning

### The Value of Research on Expert Teachers in Naturalistic Settings

Almost all of the recent research on teaching based on learning from a constraints dynamical framework has focused implicitly or explicitly on discovery learning tasks for developing children’s skill performance techniques under different constraints using the measurement tools of motor learning and development. The experimental examples we draw on in this paper are from research in naturalistic settings using qualitative descriptive methodologies (Denzin and Lincoln, 2011). The context for learning in school-based physical education can be viewed as typically impossible to sufficiently control experimentally, but offers opportunities to provide detailed descriptive accounts of teaching particular subject matter based on student learning.

In this context is Schön’s (1983) admonition that to understand professional practice, including both teaching and learning, researchers need to study more than the high hard ground that scientific research offers but also the lowland swamps of practice in naturalistic settings where problems are complex, ill structured, and not under researcher control. Relatedly, Shulman (1987) spearheaded the study of expert teachers to understand how they taught a subject matter in relation to how students most successfully learned that subject matter. This approach includes finding which aspects of the subject matter are difficult to learn, likely responses of novices that either inhibit or facilitate learning, and how to further develop skills for children at a particular developmental level (Rovegno, 1991).

The curriculum and instruction field has embraced qualitative methodologies to examine teachers’ teaching, beliefs, and knowledge, and students’ learning in intact classes in school settings (Pope, 2006; Denzin and Lincoln, 2011). The research on expert teachers we draw from in this paper were based on Schon and Shulman’s influential ideas.

### Criteria for Selecting Expert Teachers

The studies drawn on here from the Rovegno research program used Berliner’s (1986) criteria to identify expert teachers. (Chen and Rovegno, 2000; Rovegno et al., 2001, 2003; Chen, 2002; Chen et al., 2003, 2012; Rovegno, 2007, 2008). First, was demonstrated excellence in teaching. Expert teachers are typically known as such in their schools and communities and we initially sought recommendations from teachers, principals, and colleagues. These teachers were then recruited by university teacher educators to demonstrate lessons and work with university students in early field experiences and student teaching. Thus, their teaching style and effectiveness were known to the investigators. Second, accomplished teachers were sought who fulfilled some of the following criteria: awards for their teaching at the school, state, and/or national level and publications, or multiple presentations in state and national conferences. Third, highly effective teachers were recruited based on their students performing at least one and often two grade levels higher than the average teacher in similar school contexts. Fourth, the studies required teachers who were committed to what is broadly known as inquiry-oriented approaches under many different names such as a movement approach, movement education, skill-theme approach, problem solving, discovery learning, and *Every Child a Winner* (Rockett and Owens, 1977).

### How the Experts Taught Using Guided Discovery

All of the expert teachers of physical education used an inquiry-oriented approach that included scaffolding, explicit instruction, and group work during discovery tasks (Chen and Rovegno, 2000; Rovegno et al., 2001, 2003; Chen, 2002; Chen et al., 2003, 2012; Rovegno, 2007, 2008). This was a significant observation that corresponds with the large body of classroom research. Darling-Hammond et al. (2020, p. 100) summarized that productive instruction includes: “Inquiry as a major learning strategy, thoughtfully interwoven with explicit instruction and well-scaffolded opportunities to practice and apply learning; well-designed collaborative learning opportunities that encourage students to question, explain, and elaborate their thoughts and co-construct solutions.”

The expert teachers all explicitly taught or constrained tasks so that the class learned the exploration and group problem solving processes that were used in their guided discovery learning tasks. In addition, they set an inquiry-oriented environment by continually asking questions. The teachers gave feedback and instructions in the form of a question. They asked questions about why a technique, tactic, or game rule worked or was a problem, how they discovered or perceived the need for a particular tactic, what they looked for or what they saw in the environment, how they might expand on what they learned, or what might be other options they could explore. In addition, students shared what they discovered, such as what performance technique they found successful or what tactic helped them on offense and defense. Almost all of the teachers included groups designing games during the study.

After setting a discovery task, the teachers did not simply wait for children to figure out solutions on their own. They all attended to how children were exploring, their problems and successes, and decided when and how to intervene. The highly experienced teacher working with 7-year-olds and having taught the unit multiple times, knew the typical developmental patterns and scaffolded the game design process from the start using multiple techniques such as educating attention and manipulating constraints so children explored throwing from different distances at different sized targets, and using different equipment to build targets (Chen et al., 2012). Another gave brief instructions to 10-year-olds to design a game and then dealt with problems when they arose. One group had problems with fouling because they had not set any rules against fouling nor consequences for breaking those rules (Rovegno, 2007). She stopped the game and asked the group to identify the problem and told them to come up with a solution that everyone thought was fair. Regardless of the extent, all of the experts taught the process of working with partners and groups for the exploration and problem-solving process part of discovery learning tasks.

In discovery learning tasks, children are asked to explore different ways to vary a core developmental skill. Varying the movement pattern starts the process of generalizing the skill by applying movement elements such as pathways, levels, and directions using creative processes (see Table 1). Thus, in keeping with research in other subjects the teachers taught content and the related processes together in authentic contexts (Resnick and Williams-Hall, 1998). If processes are taught separately, it is anticipated that there is little transfer to domain specific knowledge.

The theoretical basis for the exploration and discovery processes was Torrance’s (1962) original four elements of creativity: fluency, flexibility, originality, and elaboration. For her research and teacher development program, Schlicter (1986) translated Torrance’s language for use across subject areas with elementary age children of all grade and ability levels with the goal for all children to increase their creative capabilities. The four processes are

1. Generate as many responses as you can (fluency).
2. Generate as many different responses as you can discover (flexibility).
3. Come up with some unusual ideas and test them out (originality).
4. Modify your ideas adding details (elaborate) to see if you can improve them.

These four processes increase the variety of movement patterns within task constraints and thus help develop generality of important game, dance, and gymnastics skills, for example, developing dribbling at different speeds and on different pathways, leaping making a variety of shapes, and balancing on different body parts in different shapes. Critical to our discussion of generality is that the generation of many, different, unusual and elaborated responses occur while children are moving.

Orth et al. (2017) described creative actions as emerging from adaptations to the relations among individual, task, and environmental constraints and are not the product of sitting, thinking and describing creative responses and then trying them out in practice. Our approach, in keeping with the goals of elementary school education differs from Orth et al. (2017) as we define developing creative capabilities and responses more broadly as goals appropriate for all children. We agree with their claims that the creative process in our field is one in which variations emerge in response to constraints and are not a top down cognitive process. The key is to keep children moving as they explore and work through a discovery tasks.

Teaching the exploration process to novices of any age during discovery learning tasks most often requires the practitioner to scaffold the process (Chen and Cone, 2003; Chen et al., 2012). To scaffold, the teacher provides a temporary structure (constraint) that assists or supports the child to successfully perform the skill or process he or she could not do independently (Vygotsky, 1978). Examples of scaffolds (temporary task constraints) include spotting in gymnastics together with equipment such as floatation devices for swimming and batting Tees for baseball. The goal is to remove the scaffolding once the child no longer needs the support.

One critical time for teachers to guide beginners while they are moving is when they are first learning how to explore. This guidance can simply be the teacher driving the pace of exploration by watching the class and when they slow down their exploration attempts or even stop to think, force the time between exploration attempts to be shorter saying, in a dance lesson for example, find another shape, and another, and another, and another, etc. Try to make a different shape each time, try a wide shape, now twisted, now narrow, now wide again, now straight, etc. which keeps children or even adult novices moving continuously at a reasonable pace simply trying different shapes without criticizing or planning or pondering their responses. Movement variety that results from novices exploring with guidance is emergent from the task constraints set by the pace of the teacher.

### Task-Relevant Augmented Information

All of the teachers intervened and provided explicit information or asked questions until the children could provide explicit information about skill techniques, tactical and game structure affordances, and what to look for in the environment. Regardless of whether the teachers discussed explicit information in the closure, at the start of the next lesson, or when the occasion presented, they eventually made discoveries explicit so that all children heard what other children had learned and could apply the skill, technique, or tactic to their own work. This general outcome is consistent with the view that teacher guidance during discovery learning plays an important role in discovery learning (Darling-Hammond et al., 2020).

The more open and challenging question is what the nature of the augmented information should be for the learner within a discovery framework. Our approach is drawn from Newell (1991, 1996) where the relevant augmented information depends on the kind of change in movement form and outcome that is pursued by the learner/teacher searching the perceptual-motor work space for task relevant solutions (Newell, 1986; Newell et al., 1989). Thus, the relevance of particular forms of augmented information will depend on the task and the skill level of the learner and the kind of change anticipated in movement form and outcome. This is because the effectiveness of inducing different kinds of change/modification in movement execution and outcome is differentially mediated by different classes of information such as: augmented verbal information, demonstrations, knowledge of results, and transition information. Augmented information channels the learner’s search strategy for mapping perception and action within and between trials in a probabilistic way. Thus, the nature of the relevant augmented information for the learner will change over the time course of practice and learning a particular skill.

In addition, because students are still learning the generality and specificity of movement skills teachers often provide guidance while children are practicing a discovery task. Teacher guidance while children are moving can be effective to guide the observation of environmental affordances including:

• the relations among individual capabilities and potential actions,
• current conditions or constraints, and
• potential conditions resulting from current conditions.

Guiding children while they are moving can help them focus their attention on the task goal such as send a pass that is catchable for the receiver. Or, teachers can cue an external focus on results, such as to hit the ball between the cones, focus on a point in the center of the cones, and work on hitting the ball there. In addition, teachers can focus on the technique most critical at the children’s developmental level such as with novice five year olds pushing with the finger pads to keep the ball under your control when learning to dribble with the hand rather than slapping the ball with the palms and losing control.

In addition, motor skills and tactics have a temporal component. When teaching children how to send a lead pass to a teammate trying to get free from a defender the teacher cues the passer to look for the moment when their teammate is traveling at a speed that is faster than the defender, and you can see their hands start to get ahead of the defenders. Judge when their hands are about to be free and send the pass into the space where they will be in another second.

## Generalization and Specificity

The generality and specificity of skill and motor skills has been viewed since at least Thorndike’s theory of identical elements on a continuum of the *similarity* between the respective perceptual-motor tasks where transfer is sought (Thorndike and Woodworth, 1901). A central question in this view becomes: what are the elements or what is the dimension on which to consider similarity and the resultant transfer of learning and performance of one task to another (Holding, 1965)? Is transfer to be sought in terms of one of the various dimensions of control that have been proposed: the effectors used within and between limbs (Stöckel and Wang, 2011), stimulus-response compatibility (Osgood, 1949), the relative timing of the movement (Schmidt, 1975); or attractor dynamics (Kelso, 1995)?

In considering dimensions of control, transfer can be positive, negative, or neutral and that it is also influenced by the order and time course of the respective task practice in developmental time making experiments on the generalization/specificity issue expensive to run and difficult to interpret. It is not surprising then that the persistence of the transfer effects is not well understood given the complexity of experimental designs needed to test adequately the hypotheses of the generality and specificity of children’s motor development. We do not know the “savings” time (if any) for practice of the benefit of generalization and whether this is trivial or meaningful to the general cost/benefit ratio of practice time/learning (Newell, 2020). This represents a substantial gap in our understanding of transfer and generalization of core developmental activities given their central place in motor learning and development.

Nevertheless, it is apparent that the motor development and motor learning literatures have advanced contrasting conclusions to the question of generality or specificity of motor skills In doing so they have pursued related but different theoretical approaches to the problem. Not only have the domains emphasized different age groups learning and performing different tasks but they have interpreted the problem through distinct aspects of the word skill. Motor development has focused on skills as tasks (as a noun) and the transfer between them at the task movement pattern level as a function of development and task type. Motor learning has addressed the question through skill (as an adjective) and the relation of the performance/skill level of the individual across different motor tasks. Although, the approaches of motor learning and development to the generality/specificity issue have been different, they are not, however, necessarily incompatible.

The emphasis here on children learning open ball game skills reveals that to some greater or lesser degree both generality *and* specificity of skills are required to play the respective game. The context of games channels the child to learn a set of individual motor skills that are subsequently used in different facets of the game. It is also the case that game contexts can induce a movement pattern and outcome that has not been practiced or performed previously. Thus, invasion games naturally lead to the complementarity of generality and specificity in that both movement pattern technique and the variability of the movement pattern in executing game tactics are needed attributes of performance.

An example of a combined generality and specificity effect is the e special (Keetch et al., 2005) where the probability of success in the closed skill of basketball shooting from the free throw line is proportionally higher than the linear scaling effects of a broader range of distances to the basket would predict. This finding is consistent with a specific scaling practice effect embedded in a more general landscape of movement dynamics, but other accounts are still empirical questions. Moreover, this is unlikely to be the only closed skill example of the complementarity of generality *and* specificity.

A key issue in unraveling the problem is the frame of reference and determination of the essential variables for movement coordination, control and skill (Newell, 1985). The hypothesis from the coordinative structure theory that what generalizes is the qualitative structure of the coordination mode (essential variables) whereas the quantitative properties of limb motions (non-essential variables – Gel’fand and Tsetlin, 1962; Newell et al., 1989; Kelso, 1995) show more transient generalizability across tasks and contexts. This hypothesis is consistent with the observation that the learned qualitative properties of the coordination pattern can be preserved over significant retention intervals (Nourrit-Lucas et al., 2013; Newell and Liu, 2020). That is, once learned, people do not forget how to ride a bicycle or swim although there is a shorter adaptive time scale of change for the emergence of the movement coordination pattern. The distinction of the essential (collective)/non-essential variables provides a framework to reconsider the different time scales of change in the learning, retention and transfer of motor skills together with their generality and specificity.

### Generality and Specificity in Teaching

Just as there is no incompatibility between generality and specificity theoretically, there was no incompatibility, conflict, or question on this issue among the expert teachers. All of the expert teachers used guided discovery tasks to teach children to generalize the basic coordination patterns to match the many varied ways the particular skill is performed in the sport or sports related to the core developmental skill. The task content of generality and specificity is drawn from the skills and movement elements of the Laban (1948) framework adapted for educational games (see Table 1).

As children worked on this generalizability, they also worked on specific skill techniques. For example, children would explore dribbling at different levels and speeds while attending to the changing distance between themselves and a defender attempting to steal the ball. The performance technique they would practice would be to dribble at a medium level at fast speeds and at a lower level when the defender was close.

In their teaching of generalized movement patterns, all of the expert teachers taught basic tactics and linked these to both movement variety and performance techniques. With 8- and 9-year-old children they used simple game-like tasks in which students explored tactics to avoid being constrained by a boundary and dribbling to get around a defender to score (Chen et al., 2003; Rovegno et al., 2003). Moreover, they linked these tactics to both movement variety and performance techniques. For example, dribble to the front side with your body between the ball and defender was linked to preventing a defender from stealing the ball.

Thus, almost from the start of teaching core developmental skills the eventual game context was discussed and aspects of it progressively integrated in lessons. Variability of the movement pattern and tactics are key attributes of game skills. All game and game-like tasks have tactics and tactical affordances, consequently, the tactical elements of the game environment need to be incorporated into guided discovery learning.

## Progression for Skills and Tactics

With the development and promotion of TGfU for secondary physical education, tactics and progression have been topics of increasing interest at the secondary school level. In TGfU students first learn through guided discovery or problem-solving tasks the tactical reasons when and why a particular skill is performed in a particular tactical way (Bunker and Thorpe, 1982). For example, a drop shot in badminton is quite useful when the opponents are back by the end line. Students then explore and practice the tactic and the skill together. The skill techniques are also learned but within the tactical tasks (Hastie and Mesquite, 2017). Essentially, TGfU tasks start with the tactics the game environment presents and students learn skills from within this tactical framework. The debate as to whether to teach skills before tactics or the reverse inspired research comparing the two approaches but the results were equivocal (Oslin and Mitchell, 2006). The approaches are difficult to compare because there can be little time, within one lesson, between the teaching of tactics and skills so any difference might not be meaningful in regards to the time course of learning.

At the elementary school level, the expert teachers all followed a basic progression for teaching invasion game content that evolved within movement approaches: (i) developing the basic coordination pattern, (ii) generalizing the basic coordination patterns and learning rudimentary tactics and game structures, and (iii) playing modified games. Teachers moved back and forth between levels based on the content and capabilities of the children in the class. Thus, the progression is not a lock-step linear process.

### Level 1: Developing the Basic Coordination Pattern

In the studies of expert, inquiry-oriented practitioners there were some teachers for whom data were available on their teaching of kindergarten and first grade children. We observed those teachers and asked others how they began to teach the core developmental skills. Every teacher reported they began when children entered school and taught the basic coordination pattern using discovery learning tasks. For example, a series of tasks having young children explore striking a variety of balls and weighted balloons with different body parts and paddles in an upward direction is a set of discovery learning tasks used by one expert (Rovegno, 2007). These are common striking tasks in all of the approaches taught by the teachers.

The task helps children learn to strike with different body parts, which can generalize to volleyball and kicking skills. The task also supports discovering the specific technique principles to get the body part or paddle under the ball, with the striking surface facing the upward direction, and the direction of the swing or hit moving also in an upward direction. These are valuable technical principles to discover that cross multiple perceptual-motor skills. Thus, the task works for developing generalizability of the movement pattern and specificity of a performance technique that is also anticipated to generalize to other similar skills.

### Level 2: Generalizing the Basic Coordination Patterns and Learning Rudimentary Tactical and Game Structure Affordances

As discussed earlier, all of the expert teachers began using discovery learning task constraints to generalize the coordination pattern as soon as children had rudimentary control of the ball most of the time even without many or anybody components showing the mature technique (Rovegno et al., 2001, 2003; Chen, 2002; Chen et al., 2003, 2012; Rovegno, 2007). They worked on movement variety and specific techniques and linked both to the tactical awareness of the related sport and what they perceive in this environment. For example, with hand dribbling they had the children explore to discover how varying the pathway (straight, curved, and angular) would work against a defender and what they needed to perceive to know which pathways to use and in which situations.

Thus, experts taught children to generalize movement patterns because these patterns are the bases for the open skills of sports. At the same time, they taught specific dribbling techniques associated with the sport of basketball. Regardless of the game or game-like task a practitioner sets the context to have tactical affordances. These affordances require children to discover and then attend to the perceptual information available, understand potential tactical responses and movement patterns required by those tactics, and then respond appropriately.

### Level 3: Playing Modified Games

The third level of the progression from skills to game play at the elementary school is playing modified games. Modified games have long been a well-accepted way to teach students different aspects of game play. For the expert teachers, modified games provided the same opportunities for discovery learning as level 1 and 2 tasks. Once children knew at a basic level how to design a game, teachers continued having students design some game elements such as the boundaries, rules for safety and fairness, and consequences for breaking rules.

In addition to tactics, they set tasks to discover game structures and their impact on game play and tactics. Bunker and Thorpe (1982) were the first to promote the importance of appreciating game structures. Bandhauer, one of the expert teachers, designed a progression for eight-year-olds for breaking down game appreciation and teaching children how to design games for discovering the meaning and tactical impact of the most basic game structures such as boundaries, rules, consequences for breaking rules, and scoring goals (Bandhauer and Rovegno, 1992). Discovering the impact of game structures is often as important as learning basic tactics in game play, especially for novices with little game play experiences. Although designed for eight-year-olds and at the most basic level of game play using tag, this progression clearly illustrates how tasks can be manipulated to form a cohesive, guided discovery progression for facilitating learning about (i) the different effects on tactical affordances resulting from different game structure designs, in this case, different boundary sizes; (ii) the related boundary tactics; and (iii) the principles that guide tactical boundary use in game play.

This progression uses the invasion game of tag. Tag is not constrained by individual skill levels in passing and receiving balls. Thus, the tasks channel students to focus on perceiving how boundaries affect game affordances and rudimentary boundary tactics without the demands of ball control.

### An Example of a Progression of Task Constraints for Teaching Game Structures

1. In a very large space have children in groups of one tagger and four to five runners play tag with no boundaries. After several rounds, ask children to discuss what was wrong with the game. They quickly discover how the lack of boundaries made it impossible for the tagger to touch anyone.
2. Then have them set boundaries that are too small and play several rounds again and ask them to identify problems they encountered. They discover small boundaries favor the tagger and are not fair or fun.
3. Next, have children design perfect-sized boundaries that afford fairness for offense and defense. Have them play a few rounds and discuss whether they need to modify their boundaries and play again. This play-discuss-play cycle continues throughout the unit. The practitioner’s role is to ask questions about what they discovered, how they used the boundaries, and how they might expand on their discoveries.
4. The next step is to design a team tag game with rules for safety and fairness, and scoring goals. Have them play and discuss their game until they are satisfied they have a good game.
5. Now shift the focus to discovering the tactical affordances that boundaries present for both the offense (the runners) and the defense (the taggers). Discovering how defenders can perceive boundaries as constraints on the space and thus the movement of offensive players, is a tactical principle used in all invasion games and one that practitioners’ can review and teach for transfer in other sport units. Without understanding the tactical meanings of boundaries, rules to stay in bounds are simply one more meaningless rule adults make for children to follow.

In their study of boys aged 14–16 years, Casey and Hastie (2011) showed how student designed games facilitated the development of knowledge about game play, the impact of game structures, and their understanding about factors that contribute to a playable, good game. Significantly, when both the younger and older children learn how to design games in a group, they also learn more about how to effectively use discovery and problem-solving processes while working with classmates in socially responsible ways.

### Adaptive Learning: Setting Task Difficulty That Challenges Students but Allows for Success

Critical to discovery tasks is the setting of task constraints that are within the capabilities of individuals and groups but that also challenge students to maximize self-organization of coordination patterns and skill techniques, learn to solve new tactical problems, and/or increase their repertoire of generality of the basic movement pattern of the skill.

Thus, the goal of setting task difficulty that challenges and allows for success is taking a developmental perspective and is central to sport psychology (Treasure and Roberts, 1995). This goal is similar to the principle of adaptive motor learning in which the task and environmental constraints are changed to keep the learner working at a state of difficulty that can facilitate self-organization into a more advanced movement pattern (Liu et al., 2012). If children find the tasks to be too difficult and never experience success, they lessen their efforts to achieve and can fail to develop a growth mindset (Dweck, 2007). A growth mindset means children believe that they can develop their abilities through effort and hard work and, in fact, when they have a growth mindset they achieve more (Dweck, 2007). In contrast, if they hold a fixed mindset and they don’t succeed they attribute their failure to a lack of innate, fixed abilities that they cannot change. They say, “I’m not good at sports, I don’t have athletic abilities so I can’t learn that very well. In turn, students with a fixed mindset achieve less.

Setting challenging tasks is, in part, the students’ responsibility in that they determine, typically through discovery learning, the pertinent skills, associated tactics, and relevance of differential equipment properties (size, shape, weight, compliance etc.). For example, all of the experts provided multiple sized or type of balls for working on dribbling with the hand (e.g., large, medium, and small playground balls, regulation or small basketballs) or striking a ball with the hand or a racket (light plastic balls, whiffle balls, tennis balls, and any paddle or racquet the school owned). Children were expected to experiment with different balls and discover which ball works best for them. In addition, two experts taught lessons during which children intentionally experimented with the effects of different equipment on game-like play and the techniques needed for successful play. They reported their discoveries to the class.

While having children select the equipment that works best for them accommodates for individual differences, it means the teacher must monitor the level of difficulty and intervene to ask if students have or have not met the criteria for developmentally appropriate practicing tasks that afford both challenge and success. The goal is for students to work near the far edge of their current potential development, that is, highly challenged but able with effort to perform the tactic, skill variation, or technique. Working at the far edge of their current potential development means the child can successfully transition to a more mature pattern because the relations among their current capabilities and the environmental affordances and task and environmental constraints will provoke self-organization. When students are working near the far edge of potential growth, the movement in action is typically fluctuating between stability and instability. The task is challenging to them but sometimes they have success in meeting task objectives. They are responding with a growth mindset that will more likely embrace difficult challenges.

### More Difficult Tasks Eliciting More Advanced Movement Patterns in Some Components and Regression in Other Components

A current motor development principle is that it is reasonable to expect beginners to exhibit more task-relevant patterns when they practice a skill in simple tasks and exhibit less stable patterns when practitioners increase task difficulty or complexity such as when motor skills are performed in games with defenders (Wickstrom, 1983; Roberton and Halverson, 1984; Gallahue and Ozmun, 1998). This regression in the pathway of change over time is, in essence, what Fitts and colleagues described as the progression- regression hypothesis in adult motor learning (Fitts et al., 1959; Fuchs, 1962). Namely, when a performer is put under stress in some way there is a tendency for the performance output and associated strategy to regress to earlier lower levels. More experienced performers are better able to adapt to new task demands and maintain mature movement patterns regardless of the task difficulty (Broderick and Newell, 1999). We begin with novice hand dribbling movement patterns and the impact of task constraints.

The data on 8- and 9-year-old children dribbling with the hand, showed, as predicted, that when the task constraints were more difficult, there was more regression to beginning patterns of slapping with the palm, more problems with ball control, and more regression instances when students contacted the ball with the second hand (Chen et al., 2003). More difficult tasks included dribbling at a fast speed, at a low level, on angular pathways around an opponent, performing a crossover dribble, and dribbling against an opponent.

In contrast, some tasks that were more difficult elicited the more advanced patterns of lifting the head to look up. For example, when the task was to dribble against an opponent, or at a fast speed, or in a crowded space, all of which would be safer if you were looking where you were going, there were more instances of students with their heads up looking around. The lowest skilled students did not look up unless the task constrained them to do so. Thus, task constraints for novices can have a positive or negative impact on specific skill techniques depending on the relations among task, environmental, and individual constraints and what information is presented and its timing.

One movement pattern that on the surface appears to be a regression is touching the ball with the non-dribbling hand in a crossover dribble. This second hand touch occurs when children work on increasing their speed, sharpness of angle, and sideways distance of their crossover dribble. In effect, they use their non-dribbling hand to touch the ball to scaffold for increased ball control. This is technically a “double dribble,” a violation. However, we view this action as a temporary scaffold that practitioners should not prohibit because it allows practice of a more advanced movement pattern. Learners will not use this scaffold once they have developed a more stable crossover dribble at a faster speed, wider distance, and sharper angle.

## The Perception-Action Link

In these next examples, the role of perception in action is evident, so much so that the movement pattern is driven by the need to control changes in the perceptual field (Rovegno et al., 2001). The task is to dodge to get free from a defender and cut by running on a straight pathway into an open space to receive a lead pass. The most beginning level pattern is to jump up and down or take one or two steps in one direction and then one or two steps in the other direction. A second more intermediate beginning pattern is to cut by sliding sideways on a curved pathway with the body facing the passer maintaining an equal distance the entire time. This reduces the perceptual demands. The perceptual aspects of the skill and tactic led to the curved movement pattern of the cut that emerged. The more advanced pattern is to run forward on a straight pathway across the passer thereby changing the distance from the passer the entire time and needing to catch the pass with arms reaching in front and the ball coming from the side.

**FIGURE 2:**
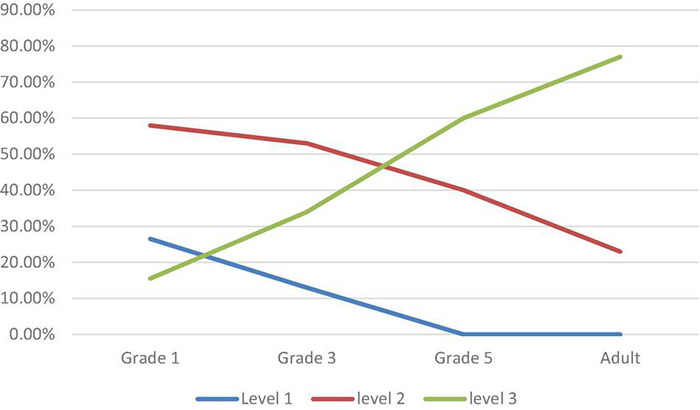
Percentage occurrence of cuts as a function of grade/Adult and Level (1-3).

Cutting on a curve was also reported in a preliminary developmental study (Rovegno, 1986) following protocols developed by Roberton et al. (1980). The study was preliminary because it was cross-sectional, not longitudinal with 6 children per grade and 3 adults. Following a typical developmental profile of how a movement pattern’s components develop across ages, the percentage of cuts was compared at each of the three component levels for four ages: first graders, third graders, fifth graders, and adults cutting and sending lead passes in groups of three. Figure 2 shows the percentage of cuts at level one (jumping behind the defender taking one to two steps to one side and then the other but never getting free) decreased from a high for grade one to lower for grades three and zero for grade five and adults. The percentage of cuts on a curve was highest in grades one and three, lower for grade five and lowest for adults. The percentage of cuts at the advanced level three cutting on a straight pathway was lowest for grade one, higher for grade three, still higher for grade five, and highest for adults.

**FIGURE 3:**
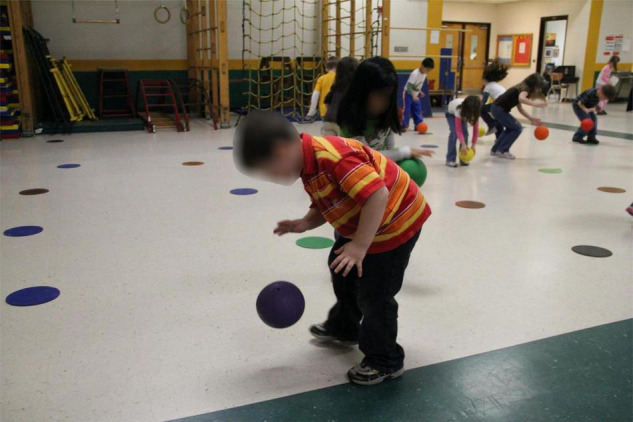
Class learning to bounce a ball. Image used with permission from John Dolly ©.

In a second example, very early in the development of dribbling with the hand, one beginning pattern is to position the head directly over the ball as it bounces (see Figure 3). This enables the child to see looming more clearly in which the coming ball gets larger as it gets nearer the eyes. Thus, the demands to perceive how high and how fast the bouncing ball is moving leads to the child assuming a less functional body posture for dribbling while traveling.

## Adapting Constraints to Provide a Task-Relevaant Learning Environment

The constraints perspective gives practitioners a framework for the adaptive manipulation of tasks and progressions to facilitate learning. In this section, we use examples from the studies to show how task, environmental, and individual constraints can sometimes fail and when modified to be more ecological can succeed in creating a successful learning environment.

Teaching is based on a cycle of setting a task and observing children’s responses and the impact of the task and environmental constraints on those responses (Barrett, 1984b). Then, subsequently interpreting their responses to determine if task and environmental constraints will lead to learning the objective of the practice session and if not, why? Finally, the teacher makes a decision as to whether to change the task or continue with the task assigned. Most often if a task is not meeting the planned objectives, the problem is not with students but with the task or the environmental constraints (e.g., the scale of the equipment, the speed and height of the ball bounce, the size of the boundaries) which are under practitioner control. Many beginning practitioners blame the students for not doing the coordination pattern or the skill technique the practitioner taught (McCaughtry and Rovegno, 2003). They fail to recognize, however, the impact of the task and environment on student performances.

One individual constraint for some children is a lack of understanding of the offensive and defensive players’ relationships. The qualitative analysis revealed that some students did not understand the relationship between the passer and receiver had consequences for how they performed skills (Rovegno et al., 2001). For example, in a task with the passer constrained to a hula hoop and a receiver dodging a defender and cutting to receive a pass, some novices capable of passing accurately tended to throw passes that were too forceful for the receiver to catch. Others heaved the ball too high with little concern for accuracy. They then blamed the receiver for not catching an inaccurate pass. The students did not recognize that the passer should want and be responsible for the receiver catching the ball because they were teammates and to throw a pass with the appropriate amount of force and height for the particular receiver.

Although many teachers initially focus on throwing and catching techniques, in the teachers’ opinions students’ beginning patterns were based on their lack of appreciation of game knowledge. That is, in a pass to a teammate the passer should want the receiver to catch the ball and to pass in such a way as to make this possible. Throwing a pass that is difficult to catch and insisting this is the fault of the receiver is a novice response that teachers did not anticipate. Consequently, they clarified the task goal and had children discover ways to send “catchable” passes (e.g., throw with less force, ask the receiver what kind of pass they need to be successful, watch the receiver carefully and throw a pass both of you are confident the receiver can catch). Focusing on catchable passes reflects an ecological and constraints perspective on teaching, that is, passing as the relations among the passer and receiver’s capabilities within the current game-like environment.

In one task to explore cutting into a space to receive a lead pass in groups of three without a defender, receivers could not apply the tactics the offense would use if there were a defender present. Receivers stood in place and waited for the passer to throw them the ball. They would catch the pass and then run with it. The teachers changed the task by adding a defender, which is often assumed to add more complexity. Instead, the defender made the environment more understandable because it was more representative of a game situation and added information critical to the novices (Gibson, 1979). With a defender, the tactic to run into an open space made sense and the affordance was more perceivable. The quality of students’ practice improved.

Another set of constraints that the teachers changed to create a better learning environment in games was the balance between offensive and defensive abilities. When the passers and receivers were less skilled at tossing and catching balls and defenders were quick and played with high intensity, the receivers could not get free and passers could not successfully pass (Rovegno et al., 2001). When defenders were slow and played with low intensity, receivers stood still and passers tossed them the ball.

The teachers responded by changing the task limiting the number of defenders to 3 vs. 1 or 3 vs. 2. This resulted in more successful passing practice. In addition, teachers had players adapt their level of defense to the level of their classmates’ offensive skills using 5 levels of defense.

1. Defender keeps his or her feet still and arms still.
2. Defender keeps feet still, but the arms can move.
3. Defenders can travel, but they have to keep their hands behind their backs.
4. Soft guarding (defenders can move arms and travel, but don’t guard with high intensity. The receiver has to work hard, but most of the time, the defender lets the receiver be successful in catching the pass).
5. Full, high-intensity guarding.

Using the appropriate levels of defense benefited receivers perceiving the affordances of open space and then cutting into one of the spaces. It benefited the passers perceptions of receivers as they got free from a defender and timing when to send the lead pass into the space ahead. When the defense was stronger, the game failed to provide a good learning environment. With a good balance between offense and defense the task constraints worked to keep the students practicing skills and learning tactics at the edge of their developmental level.

One instance in which students’ offensive abilities were stronger than their defensive ones, was when there was a change of possession. The offense quickly took off toward their goal and the defense looked lost and did not know where to move. The rules students initially chose were to change possession on the spot of a foul or immediately after a goal. Dropped balls or incomplete passes were free balls. These game structures were well beyond the capabilities of almost all of the children. The change from offense to defense elicited only beginning patterns. They did not quickly change their position in relation to teammates and opponents. When there were many inaccurate passes and dropped balls were free balls, there were too many times children pushed others to get the ball from the ground. The teachers asked students to identify the problems and possible rule changes sometimes giving suggestions such as try starting from the end line or the nearest sideline with each score or violation. No free balls. Dropped balls are a change of possession.

The amount of space for scoring goals was another task constraint that had a strong impact on whether the game was a good learning environment or not. When the students choose to use one hula hoop as a scoring goal, this made scoring too difficult because the defenders simply crowded around the goal. This is an excellent tactic for preventing goals but one that makes for a poor learning environment and a game that is not representative of the more balanced relations among offensive players and defenders in basketball or other invasion game scoring opportunities. The teachers had students discuss the problems and possible solutions including scoring at two different goals or passing to a teammate over the end line. Students had to anticipate what each choice would offer (giving the offense more options and space and consequently spreading the defense apart) and decide as a group how to solve the problems.

Constraints that provided both a challenge and successes worked well to create good learning environments. To summarize ways of maintaining task, environmental, and individual constraints in modified games the teachers posed the problem to the students. Experiment with what makes a good game for learning. Then we will discuss what you found. Along with an analysis of the children’s games, the discussion (in parentheses) revealed the following criteria were important for having a good game that provided an environment and task that allowed for students to engage productively in discovery learning tasks that worked well (Rovegno and Bandhauer, 2017).

1. The game has flow and does not stop frequently. (The rules worked for safety but didn’t stop the games a lot. It didn’t stop because we were arguing. We didn’t get confused and stop. We knew how the game worked).
2. There is balance between offense and defense capabilities and the amount of time playing in those roles. (One team did not have the ball the entire time. No one hogged the ball. Both teams got to score. It was fair).
3. All group members understood and agreed on the rules and scoring system. (We got along and knew what we were doing).
4. Task and environmental constraints ensure that students are challenged but successful (Games were fun, exciting, interesting, and not too hard so we could play the game without problems).

Learning about how to design games that were playable and worked well was also a major finding of the study with 14–16 year-old boys (Casey and Hastie, 2011). They learned how rules had an impact on the tactics and how to solve the problems that a lack of a particular rule caused. Critical to note is that the older boys were better able to identify and solve problems with game structures than the 9-10 year-olds. This is expected because the younger children were playing their first modified basketball-type games, while the older boys had many experiences with modified game play.

## Constraints on Teaching Through Guided Discovery Learning

In this paper we have outlined a framework for how the teaching of core developmental perceptual-motor skills in children can be harnessed to and guided by contemporary research on the dynamics of movement coordination, control and skill and exploratory strategies that unify learning and teaching. Our practical context was elementary school age children learning open skill invasion ball games through discovery learning. School physical education classes tend to have a more representative population of children than junior sports and a wider range of skill levels. Team ball games were our focus because in spite of their prevalence in physical activity and sport there has been generally limited research directed to the learning of open perceptual-motor skills (Poulton, 1957), by both children and adults. It was anticipated that the perceptual-motor demands of open game skills would broaden the outcomes from those established in children’s learning of closed skills. We close with brief summary remarks on a few key future issues for the learning of team ball games by young children.

### The Open Skills of Invasion Team Ball Games

Invasion games in the form of soccer, basketball and so on allow each team to seek a presence of their players in essentially any part of the bounded playing arena for that game (Almond, 1986). The open skills and tactical situations in invasion games require a sufficient level of particular skills that can be generalized in a variety of ways.

The qualitative analysis of the Rovegno research program on invasion ball games has provided a preliminary set of observations on children’s perceptual-motor skill learning of open skills and tactics. The studies showed that most children of elementary school age (5–10 years) not only have to learn the movement pattern of, for example, how to bounce or pass a ball but also how to generalize the skill in a variety of ways, and learn basic tactics about using the skill against a defender. The expert teachers uniformly brought these elements of movement pattern and game tactics together as soon as the child exhibited the rudiments of successfully bouncing a ball. Having children explore and develop the generality of dribbling through the movement elements of the Laban framework provided a powerful analytic tool for teachers and children to identify these options. These fundamental features of invasion games invite a broader consideration of whole-part-whole learning than has been predicated to date on closed perceptual-motor skills.

### Generality and Specificity

The open skill nature of team invasion ball games naturally requires the generality *and* specificity of skill and skills on the part of the player. The degree of unpredictability of open skills also assures that in practice there is not a single balance of these action properties for success. In this context, one might ask Messi, who many view as the world’s best soccer player, that if he had it all over to do again would he have spent more time in his childhood days educating his right foot and would this necessarily have been at the expense of the extraordinary ball skill he has developed with his left foot?

### Tactics, Game-Like Tasks and Modified Games

The analysis of expert teachers revealed the potential of a constraints approach and guided discovery for teaching invasion game play. The students of experts who taught the tag unit discovered for themselves blocking and setting screens and picks, and one on one and zone defense. In all of the dribbling units that included games, children used tactics without prompting, putting their bodies between the ball and defender and defenders using boundaries to constrain the offense. The working assumption is that there is generality in tactics across the different invasion games.

While promising, the analysis also revealed the special challenges of teaching young children team ball games that go beyond learning the basic relevant movement patterns to the many issues of team and game tactics. The qualitative analysis showed that learning the constraints on playing space and boundary conditions and the notion of opposing team defense and offense were as or more challenging than learning to coordinate the bouncing of a ball. In game-like tasks and games, tactics and skills are relational. Children need to perceive the relations among teammates and defenders, for example, perceiving how close the defender is to the receiver and the abilities of the receiver to catch a pass and their own ability to throw an accurate pass at the particular distance that is presented.

The analysis of task constraints that did not work well and were consequently modified by the teachers with the addition of defenders revealed that defenders did not add complexity to tasks, rather they added critical information that made the task and tactics meaningful. Children could more easily see how and why a tactic worked to further offensive play. In addition, the tactical affordances that are always present became easier for the children to perceive because the relations among offensive and defensive players were more evident.

Designing multiple modified game task and environmental constraints that worked well to facilitate learning with less skilled groups needing different task constraints than more skilled groups was most difficult for teachers. This is in part because all but two expert teachers taught classes with over 40 children. Critical was controlling the level of defensive skill and intensity to match the abilities of children in offensive game play. The relations needed to be balanced so that the game had similar characteristics of the related sport and was fair, enjoyable, and a learning experience for all players.

### Adaptive Dynamics

The primary constraints to action in this learning-teaching developmental framework should be coordinated so as to keep the self-organization of skill development (movement pattern and tactics) continually evolving, while preserving the child’s motivation and enjoyment for the expanding repertoire and performance capacity of his/her perceptual-motor skills. Changing a control parameter(s) as a task constraint should be aimed progressively to keep the task difficulty as an achievable challenge.

### Guided Discovery Learning

The analysis by synthesis revealed a number of features of contemporary movement coordination, control and skill that fit well with the general tenets of the guided discovery learning construct (Bruner, 1961; Vereijken and Whiting, 1990; Vereijken, 1991; Darling-Hammond et al., 2020). The open skill nature of team ball games enhances the relevance of inducing self-organized search strategies to movement solutions for both the generality *and* specificity of the child’s evolving movement repertoire, and the tactical use of these open skills in games or game-like situations. Teachers through progressively manipulating the environmental and task constraints channel the search strategies and discovery learning of the child.

The studies of expert teachers showed that using guided discovery can elicit more advanced performance techniques, generalization of core developmental skills and tactical responses to invasion game play in novices just beginning to learn this content. The constraints of the individual, environment and task coalesce to provide a context for motor learning and performance (Newell, 1986). This framework of constraints can be and was modified by the teachers including through the addition of oral communication of explicit information, feedback, and scaffolding of the discovery process and complex information. Different skill levels in different tasks need the support of different types and frequency of augmented information for effective and efficient learning (Newell, 1996). Thus, the nature of the augmented information along with the task and environment manipulations from the instructor, differentially influences the search strategy and resulting level of performance outcome.

Like the research in other subjects, the teachers used inquiry-oriented *guided* discovery in which they not only set discovery tasks but also gave explicit information, scaffolded the discovery process, and provided instruction and feedback. This finding is significant because it counters the many misconceptions held by teachers about inquiry-oriented approaches, such as “Don’t tell children what to do, don’t intervene, just let them get it all through discovery.”

### Qualitative Observations in Natural Settings and Quantitative Measures in Experimental Settings

Qualitative observations and experimental measures in school settings bring different strengths and limitations to the analysis. One can view the former as being a preliminary first pass at the problem to inform the latter and the implementation of a cutting-edge experimental program. On the other hand, without the injection of substantial funding for a broad program of quality experimental studies in children’s motor development (as in STEM and Reading) the tailoring of qualitative observational analysis may prove to be the only or major pathway forward to enhance our understanding of the development of children’s movement skills in physical activity.

## Author Contributions

Both authors listed have made a substantial, direct, and intellectual contribution to the work, and approved it for publication.

## Conflict of Interest

The authors declare that the research was conducted in the absence of any commercial or financial relationships that could be construed as a potential conflict of interest.

## Publisher’s Note

All claims expressed in this article are solely those of the authors and do not necessarily represent those of their affiliated organizations, or those of the publisher, the editors and the reviewers. Any product that may be evaluated in this article, or claim that may be made by its manufacturer, is not guaranteed or endorsed by the publisher.
